# Contribution of natural food environments to nutritional intake and biomarker status: insights from the women of indigenous santhal communities of Jharkhand, India

**DOI:** 10.1186/s40795-023-00669-1

**Published:** 2023-01-27

**Authors:** Archna Singh, Ayushi Dhasmana, Ashish Bandhu, Ridhima Kapoor, Sivasankar Baalasubramanian, Suparna Ghosh-Jerath

**Affiliations:** 1 Department of Biochemistry, All India Institute of Medical Sciences, New Delhi, India; 2 The George Institute for Global Health, 308, Third Floor, Elegance Tower, Plot No.8, Jasola District Centre, New Delhi, 110025 India; 3 School of Institute of Health Management Research, IIHMR University, Jaipur, India; 4 Immunitas Biosciences Private Limited, Bangalore, India

**Keywords:** Indigenous communities, Santhal community, Natural food environments, Kitchen garden, Micronutrient status, Inflammatory biomarkers

## Abstract

**Background:**

Many indigenous communities reside in biodiverse environments replete with natural food sources but show ​poor access and utilization.

**Methods:**

To understand the links between indigenous food access, dietary intakes, and biomarkers, we conducted a cross-sectional study among women of the Santhal Community (*n* = 211) from 17 villages in the Godda district of Jharkhand, India. Survey methods included household surveys, dietary intake assessment (24 HDR) and micronutrient and inflammatory biomarkers' estimation.

**Results:**

The diversity in access to foods from different natural sources expressed as Food access diversity index was low. This led to poor consumption and thus a low Minimum Dietary Diversity. The mean nutrient intake was less than the estimated average requirement for all nutrients. Women with higher dietary diversity scores had higher nutrient intakes. Thiamine and calcium intakes were significantly higher in women consuming indigenous foods than non-consumers. One-fourth of the women had elevated levels of inflammatory biomarkers. The prevalence of iron deficiency was approximately 70%. Vitamin A insufficiency (measured as retinol-binding protein) was observed in around 33.6% women, while 28.4% were deficient. Household access to natural food sources was associated with specific biomarkers. The access to kitchen garden (*baari*) was positively associated with retinol-binding protein levels and negatively with inflammatory biomarkers, while access to ponds was positively associated with ferritin levels.

**Conclusion:**

The findings highlight the role of access to diverse natural foods resources, including indigenous foods, for improving nutrition security in indigenous communities. Nutrition and health programs promoting indigenous food sources should include the assessment of biomarkers for effective monitoring and surveillance.

**Supplementary Information:**

The online version contains supplementary material available at 10.1186/s40795-023-00669-1.

## Background

Micronutrient deficiency or hidden hunger is a crucial global health issue affecting more than 2 billion people worldwide [1]. It has many adverse health outcomes, including poor physical and mental development in children and loss of productivity and human potential [2]. Among the developing nations, the simultaneous manifestation of undernutrition, overnutrition, and micronutrient deficiency, also known as the triple burden of malnutrition, continues at high levels, and the progress in tackling the same has been unacceptably slow [3]. It leads to a vicious cycle of underdevelopment [4], which disproportionately affects vulnerable populations consisting of young children, adolescent girls, pregnant and lactating women, economically marginalized people, and indigenous populations [5, 6]. The current rates of anemia in India are alarming with the prevalence being 57% in women of reproductive age (WRA) and 67.1% in children 6 to 59 months [7]. If the current trends continue, India will have an excess of 22·8% anemia prevalence among the WRA group relative to WHO and UNICEF target of 2030, reiterating the need for more significant improvement rates [8].

India is home to 104 million indigenous people spread across 705 indigenous communities, constituting 8.6% of the country's population. These are designated as "Scheduled Tribes" by article 342 of the Indian Constitution [9]. Compared to their non-indigenous counterparts, the nutritional status of the indigenous population is significantly poorer. The prevalence of chronic energy deficiency (CED) and anemia in WRA are higher among the indigenous population (31.7% and 59.9%, respectively) as compared to the corresponding national Figs. (23% and 53.1%, respectively) [10]. This high prevalence of malnutrition is even though indigenous people hold guardianship of 80% of the global biodiversity and possess traditional ecological knowledge^1^ (TEK) about their natural food environment,^2^ consisting of several indigenous foods (IFs), which are potentially rich sources of nutrition [11–13]. Indigenous foods are derived from plants, animals and fungi species, occurring in a particular geographic place accessed as part of traditional food systems from the natural food environment i.e., wild and cultivated food environments [14]. The rich TEK about the natural food environment can contribute to dietary diversity and can effectively address malnutrition [15].

Many programs for the development and protection of indigenous people exist globally. Likewise, in India, development of indigenous people has been at the center stage of every 5-year plan by the government. However, a wide gap exists in achieving desired nutrition and health indicators [16] owing to multiple factors such as food insecurity due to uncertainty of food supply; primitive agricultural practices; seasonal fluctuations and climatic disruptions; changing employment patterns and geographical remoteness [6, 17]. Other factors include socioeconomic vulnerability, poor household conditions, and inadequate public health infrastructure [16, 18].

National surveys play a crucial role in informing, designing, and implementing sound public health programs and monitoring the progress of ongoing programs, however these data sources rely primarily on self-reported dietary intake data [19]. More recently, the Comprehensive National Nutritional Survey (CNNS) 2016–18 provided robust nationally representative data by assessing biomarkers for micronutrient deficiencies and non-communicable diseases [19]. However, other than the CNNS, only a few studies have reported information on nutritional biomarkers in India owing to technical and logistic limitations such as special needs for specimen collection, handling, transport, and inadequate local infrastructure for testing. In addition, biomarker surveys have low participation rate due to the invasive nature of sampling, making them less feasible in large-scale nutrition surveys [20, 21].

Biomarkers of nutritional intake objectively and precisely quantify nutritional status and are not subject to measurement errors from recall bias and misreporting. They can be measured in multiple tissue types and help to validate traditional dietary assessment methods [20, 22]. Hence combining dietary biomarker data with self-reported data can strengthen the link between diet and disease and has potential to assist in the precise assessment of dietary intake [23, 24].

Jharkhand, a central-eastern state of India, is one of the most biodiverse regions having a large indigenous population constituting 26.3 percent of the state's total population. In Jharkhand, Santhal is the most populous indigenous community representing 34 percent of the total ST population, and is spread over vast areas of Giridih, Dumka, Purbi Singhbhum, Pakaur, and Sahibganj districts. The majority of the Santhals in Jharkhand are cultivators (48.6%) and agricultural laborers (38.4%) [25]. Many studies have documented a high prevalence of CED, undernutrition, and anemia among WRA, adult men, and children in the Santhal community residing in Jharkhand, Bihar, Orissa, and West Bengal [26–29]. Our previous studies have also indicated that Santhals have access to a biodiverse agroforestry environment consisting of natural food environment (forests, ponds/rivers, open fields) and cultivated food environment (agricultural land, kitchen gardens) consisting of around 103 diverse types of indigenous foods including a number of green leafy vegetables, other vegetables, fruits and meat and meat products [30].

The work described in this paper is a part of a larger project that examined the contribution of IF consumption towards dietary diversity and food security among WRA and children of indigenous communities of Jharkhand. This paper reports the iron and vitamin A levels along with the status of inflammation among the Santhal WRA group using selected biomarkers. Further, it explores the associations of biomarker levels with dietary intake, with a special emphasis on indigenous food intake and dietary diversity; nutritional intake; sociodemographic and economic factors; and access to different food sources.

## Methodology

The present study is part of a larger project, with the comprehensive description and methodological approaches reported elsewhere [31]. In the present paper, we report the micronutrient and inflammatory biomarker status of the Santhal women and explore their association with the dietary, indigenous food, nutrient intake, and nutritional status in the context of their socioeconomic and demographic profile and food environment characteristics.

### Study design and locale

This exploratory cross-sectional study was conducted in July–August 2018 in seventeen selected villages of Godda district of Jharkhand. The population of Santhal community in Godda district was 224,068 as per the census conducted in 2011 [32].

### Sampling framework and study population

A two-stage cluster sampling was used wherein blocks (an administrative subdivision of a district) were purposively chosen (Sundarpahari, Boarijor, Pathargama, and Poriyahat), followed by random selection of a total of 17 villages (with a high concentration of Santhal population) using probability proportional to size (PPS) sampling from the selected blocks. This was followed by the second stage, in which a house listing of Santhal households (HH) was carried out, and a sampling frame of all eligible HHs was constructed. Based on the larger objective of the project, all HHs with at least one non-pregnant WRA group (15–49 years) and one child (6 to 54 months) were selected for the study. This paper reports the observations on all eligible women who fulfilled the inclusion criteria (age 18–49 years, not pregnant at the time of the study, had no self-reported morbidity that affected current dietary intake, and gave consent for participation). If more than one eligible woman was present in a HH, one woman was randomly selected using a Kish table [33].

### Sample size calculation

The sample size for the present study is based on the objective of the larger study, which aimed at assessing the differences in micronutrient intake between IF consumers and IF non-consumers. The present study, however, did not analyze the data on the basis of IF consumers and non-consumers. Iron intake data from our previous study on same indigenous community [34] was used for sample size calculation based on the difference in mean dietary intake of iron of 4 mg/day (between the consumer of IFs and non-consumers) with a standard deviation of 7 mg/day between the two groups. In order to detect a suggested minimum difference in iron intake (80% power and 5% level of significance), a minimum of 97 women in each group was required. A design effect of 2.0 was considered to account for the loss in precision due to cluster sampling and deviation from normality. A sample size of 194 women was derived, and all the eligible HHs identified during HH listing were covered (*n* = 327). The HH survey could be completed in 275 HHs, a dietary survey in 266 women, and blood sampling could be completed in 238 women. After data collation, cleaning, and removal of outliers, a common pool of 211 women were included for analysis.

### Study procedures

**Fig. 1:**
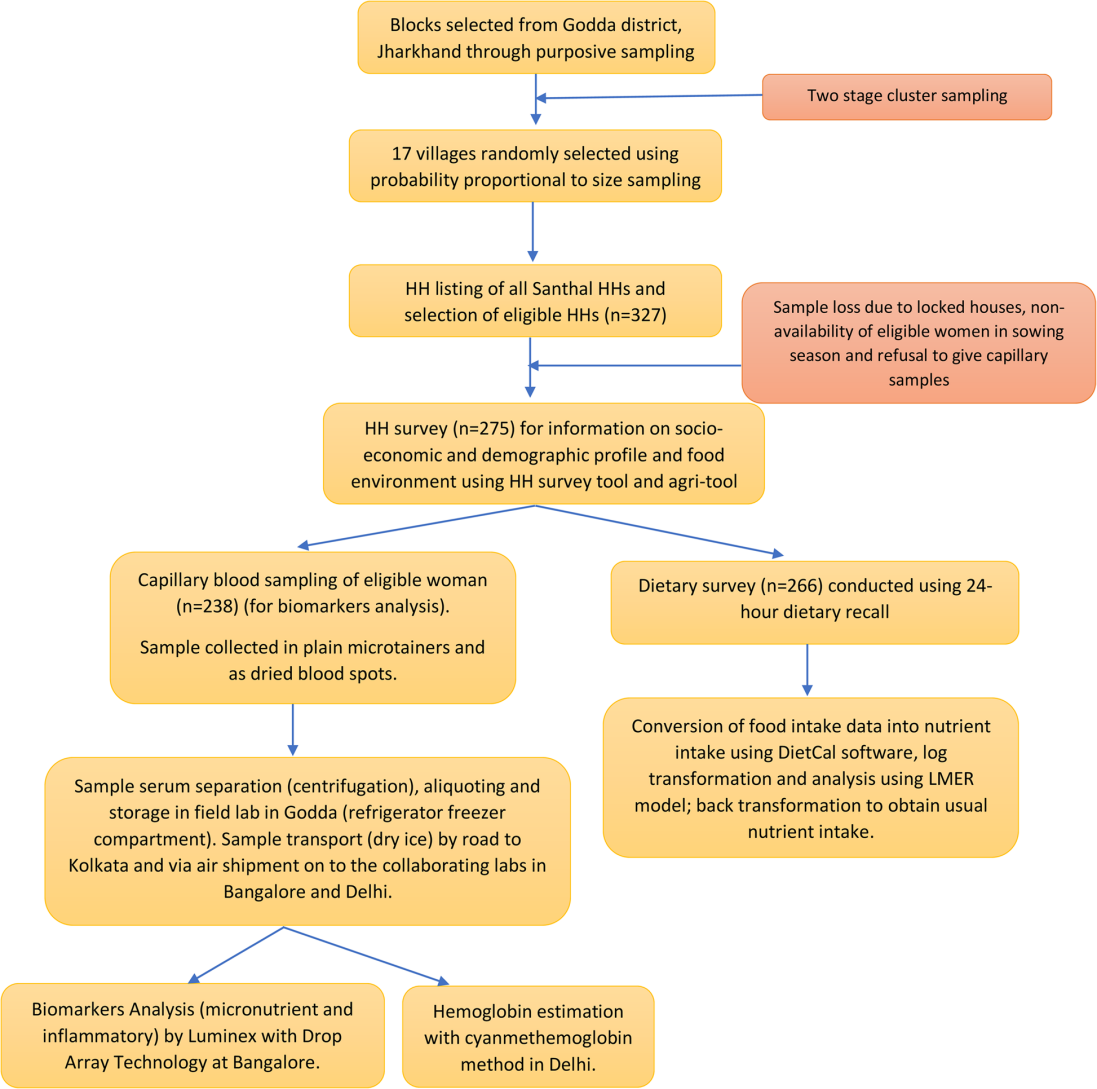
Overview of the sampling framework and study procedures

We conducted three different research activities, i.e., the Household survey, dietary survey, and collection of capillary blood samples to analyze biomarkers. An overview of the sampling framework and study procedures is given in Fig. 1.

#### Household (HH) survey

A pretested structured questionnaire developed on an electronic data capture (EDC) platform using the software CS Pro (Version 7.1) and Samsung tablets (Model SM‐T385) was administered at HH level to gather information on HH characteristics. At the HH level, any adult member of the family, preferably a woman in the HH, who had information on household food access and other HH characteristics, was administered the HH survey. This survey elicited information on socioeconomic and demographic characteristics such as age, educational and occupational status of HH members, type of house, number of rooms, presence of separate kitchen, access to water and sanitation facilities, and some other features. Besides this, information on availability and access to different food sources such as HH ownership of agricultural land and kitchen garden (*baari*), access to forests for gathering food, access to water bodies (pond/river), etc. was also collected using HH survey tool and agriculture diversity tool (agri-tool) (on a sub-sample).

#### Dietary survey

A 2-day, non-consecutive 24-h dietary recall (24 HDR) was conducted on the eligible woman from each HH to estimate nutrient intake. A food recall kit consisting of standard utensils and a food picture flipbook for portion size estimation was used and the participant was asked to recall the food items consumed in each meal over the past 24 h with their corresponding approximate amounts, the ingredients used, and the method of preparation followed for each recipe consumed. In order to minimize the error in self-reporting, a final probing was done to make the woman recall if any additional food was consumed but not reported. This information on the self-reported amounts of raw foods was used to calculate the daily intake of individual food groups. Nutritionists and nutrition interns administered a paper form after due training for dietary recalls.

#### Capillary blood collection and analysis

##### Sample collection, storage, and transport

A clinical biochemist provided training on sampling and processing procedures to the field worker and a laboratory technician. During the daily surveys, the capillary blood collection procedure included cleaning the ring finger of the non-dominant hand with an alcohol swab, pricking the finger using a lancet, collecting the blood in labeled microtainers, and storing them in an icepack lined icebox. Simultaneously, spotting of drawn blood (20 µl) on Whatman paper was also done to estimate hemoglobin from dried blood spots (DBS). At the end of each day's collection, serum of collected samples was separated by centrifugation; multiple aliquots were made and stored in cryoboxes in the freezer compartment of the refrigerator at the field site laboratory. These samples were then transported in dry ice to Kolkata via road and subsequently to the Bangalore laboratory (Certificate of incorporation no (CIN): U74900KA2015PTC079960; Start-up registration number: KBITS/SK-REGN/2016/165) and AIIMS, New Delhi via air shipment.

##### Biomarker estimation

The collected blood samples were analyzed for micronutrients and inflammatory markers- namely ferritin, soluble transferrin receptor (sTfR), Retinol Binding Protein (RBP), alpha-1-acid glycoprotein (AGP), C-Reactive Protein (CRP), and hemoglobin. An in-house assay was developed and validated for measuring these biomarkers in partnership with the laboratory at Bangalore. The method was developed using Luminex technology on a Luminex xMAP ® platform along with the drop array technology. More details have been included elsewhere [35]

##### Hemoglobin estimation

For Hemoglobin estimation, a colorimetric method based on the formation of cyanmethemoglobin (Benesphera™) was used as per manufacturers' protocol, and all DBS samples were processed in a single batch. The DBS was cut out, transferred to test tubes containing 5 ml of Drabkin's solution, and kept at room temperature (20–25°) for 6 h. After that, one mL of the Drabkin's solution was measured on a spectrophotometer at 540 nm (OD540). A standard curve was constructed using hemoglobin standards provided with the kit and used for calculating the hemoglobin concentrations in the DBS samples.

### Analysis of the study variables

#### Socioeconomic status

A HH wealth index score was obtained using principal component analysis (PCA). Variables such as type of housing, ownership of selected assets, number of rooms, presence of separate kitchen, source of electricity, access to water and sanitation facilities, and monthly expense on food were used to generate HH Wealth Index. Depending upon the quantiles of the HH wealth index score, the HHs were classified into five groups.

#### Food environment assessment

A modified and adapted version of the crop diversity index (CDI) [36] namely the Food Access Diversity Index (FADI), was developed for measuring access to diverse natural food sources. Lower diversity in production and access to foods is indicated by lower values of FADI and vice versa. It was expressed as:

$$\mathrm{FADI}=\left(\frac{\mathrm n}{\mathrm N}\right)^2$$

Where n is the total number of foods grown, gathered, or accessed and animals raised in a particular HH and N is the maximum possible number of foods grown, gathered, accessed, or raised in a specific village. Detailed information on FADI has been reported elsewhere [37].

#### Analysis of dietary intake data

##### Diet quality

The Minimum Dietary Diversity for Women (MDD-W) was calculated as per FAO and USAID guidelines[38]. It was calculated by adding the number of food groups (for food items with amounts ≥ 15 g) consumed by the women reported each day in the 2 days 24-HDR, and then the mean score for two days was calculated. Indigenous food consumption scores (IF score) were calculated from the 2 days 24-HDR by adding the number of indigenous foods from different food groups consumed by the women each day and then calculating the mean IF scores. If a woman consumed any IF over the recall period of 2 days, she got a score of ≥ 0.5 and if she did not consume any IF, she got a score of ‘0’.

##### Nutrient intake

A validated software, DietCal (Version 8.0; Profound Tech Solution), converted food intake data into nutrient intakes. As the data on nutrient intake was skewed, the box cox method was deployed to transform the distribution into a normal distribution. The transformed data was then used in the linear mixed effect regression (LMER) model, and the values thus obtained were back-transformed to calculate the 'usual nutrient intake' [39]. The estimated average requirement (EAR) cut-point approach was used to estimate the prevalence of nutrient adequacy using these usual intakes, except for iron, for which the full probability approach [40] was utilized to determine the adequacy. The reference values of EAR were taken from the latest nutrient requirement guidelines for Indians [41].

#### Analysis of biomarker levels

Table 1 provides information on cut-offs values taken and case identification based on the biomarkers. The ferritin and sTfR concentrations were adjusted for inflammation (CRP and AGP) using a linear regression model [42]. The linear regression model is used to predict the outcome variable, which is a continuous variable, given the number of predictors. In our study, ferritin was the outcome variable and predictors were CRP and AGP. Since these variables were not following normal distribution, these were log-transformed, and then a linear regression model was fitted. If CRP and AGP values are given then one can predict the value of ferritin using the given equation. The mathematical form of the model is Y = a + b1X1 + b2X2 + …. + bnXn, where, a = intercept, b1, b2, b3, …bn are regression coefficients and X1, X2, X3,..Xn are predictors. Thus, using log (CRP) and log (AGP), a linear regression model was developed to predict the log (ferritin) as the outcome. After that, the adjusted ferritin values were determined using the estimated regression coefficients in the following equation:

$$\log\left(\mathrm{ferritin}\right)\mathrm{adj}\;=\;\log\left(\mathrm{ferritin}\right)\;-\;0.209\;\ast\;\left(\log\left(\mathrm{CRP}\right)\;-\;1.00841\right)\;-\;(\left(-0.01936\right)\;\ast\;\left(\log\left(\mathrm{AGP}\right)\;-\;6.21063\right))$$

**Table 1:** Cut offs and case identification based on biomarkers

Biomarker	Cut-off values	Case identification
**Biomarkers of iron deficiency**		
Ferritin	< 30 ng/mL	Deficient iron stores
sTfR	> 2.07 µg/ml	Iron-deficient erythropoiesis
Hemoglobin	< 80 g/L	Severe anemia
	80–109 g/L	Moderate anemia
	110–119 g/L	Mild anemia
	< 120 g/L	Any anemia
**Biomarkers of Vitamin A deficiency**		
RBP	< 0.7 mmol/L	Vitamin A deficiency
	< 1.05 mmol/L	Vitamin A insufficiency
**Biomarkers of inflammation**		
CRP	> 1 mg/L	Presence of inflammation
AGP-1a	> 840 mg/L	Presence of inflammation

In order to obtain the ferritin values in ng/ml, antilog of log(ferritin)adj values were determined. Similarly, log (sTfR) was predicted deploying a linear regression model using log (AGP) and the sTfR values were obtained using the estimated regression coefficients in the following equation:

$\log\left(\mathrm{sTFR}\right)\mathrm{adj}\;=\;\log\left(\mathrm{sTFR}\right)\;-\;0.241\;\ast\;\log\left(\mathrm{AGP}\right)\;-\;6.211)$ and then antilog of $\log\left(sTFR\right)\;adj$ values was taken to obtain the sTfR values in µg/ml. As the data on all biomarkers was skewed, log transformation was deployed to transform the distribution into a normal distribution.

### Statistical analysis

Data entry and collation were done using Microsoft Excel and Stata SE version 15.1 [43]. The categorical variables were reported as frequency and percentages, whereas the continuous variables were reported as mean and standard deviation. Bivariate analysis was done using Karl Pearson correlation coefficient, t-test, and one-way ANOVA along with post hoc test for association between exposures such as nutrient intake, IF score, MDDW, sociodemographic and economic variables, access to different food sources, and the outcome as biomarkers. The associations that were statistically significant at *p*-value < 0.05 were transferred to the Linear Mixed Effects Regression (LMER) model for multivariate analysis.

### Ethical considerations

Ethical approval for the study was obtained from Institutional Ethics Committee at the Indian Institute of Public Health‐Delhi, Public Foundation of India, and The All India Institute of Medical Sciences, New Delhi. Administrative approvals were also taken from the authorities at the district level. Cluster level consent from the village leader was obtained before any data collection. Informed consent (verbal witnessed consent in case the participants could not read or write and signed non-witnessed written informed consent for literate participants) was taken before the administration of the interview, and collection of blood sample and for publication of the data. Participation in the study was voluntary and small incentives, procured from the local markets were given to the participants.

## Results

### Sociodemographic and economic profile of Santhal households

Table 2 presents the sociodemographic description of the study population (*n* = 211). The mean age of the women was 27.2 ± 7 years, with a majority having a primary or lower level of education (78.2%). The sample population was divided into five quintiles based on the HH wealth index, the mean wealth score of the lowermost, and the uppermost quintiles, were -2.8 ± 0.7 and 3.2 ± 1.4 respectively.

**Table 2:** Sociodemographic and economic profile of Santhal households, Jharkhand, India (*n* = 211)

Characteristics	n	%
**Education level of women**		
Primary or lower (till 5^th^ standard)	165	78.2
Above primary	46	21.8
**Occupation of the women**		
Engaged in unpaid household/care work	142	67.3
Shifting Cultivation/ Settled Agriculture	40	19.0
Gathering	12	5.7
Daily wager (agriculture and non agriculture)	15	7.1
Service (Government or Private)	2	0.9
**Family type**		
Nuclear	101	47.9
Joint/ extended	110	52.1
**Main source of drinking water**		
Tube well/Hand-pump	134	63.5
Well	73	34.6
River/Dam/spring /waterfall	3	1.4
Piped water	1	0.5
**Place of defecation**		
Open Field/ Jungle	193	91.5
Own Toilet	18	8.5
**Characteristics**	**Mean**	**SD**
Age of women in years	27.2	7.0
**HH wealth index**		
Lowermost quintile	-2.8	0.7
Lower middle quintile	-1.2	0.3
Middle quintile	-0.2	0.3
Upper middle quintile	1.0	0.4
Uppermost quintile	3.2	1.4

### Household access to different food sources and water and sanitation

The households reported accessing various food sources such as agricultural land (92.9%), kitchen garden (*baari*) (71.6%), water bodies (ponds and rivers) (55.5%), and forests (54.5%). The food items accessed from different food sources are listed in Table 3 and 4. The mean quantitative estimate of foods accessed from different food sources expressed in terms of FADI was 0.3 ± 0.3, which suggests low diversity in production and access to foods from the natural environment. The FADI was found to be significantly associated with the family type (nuclear v/s joint) (*p* < 0.001) with higher mean value of FADI in joint family (mean = 0.39 ± 0.32,) as compared to nuclear (mean = 0.24 ± 0.23), suggesting higher diversity in access to different foods in HHs with joint family.

**Table 3:** List of food items accessed by Santhal households from agricultural land and kitchen garden (*baari*)

S. No	Food items			Accessed from	
	**Local Name**	**Common Name**	**Scientific Name**	**Agricultural land (*n* = 196)**	***Baari*** **(*n* = 151)**
**Cereals**					
	Rice	Rice	*Oryza sativa*	180 (91.8%)	-
	Jondra/Makai	Maize	*Zea mays*	65 (33.2%)	101 (66.9%)
	Bajra	Bajra	*Pennisetum typhoideum*	4 (2.0%)	-
	Janhe/Ragi	Ragi	*Eleusine coracana*	2 (1.0%)	-
	Jowar	Sorghum	*Sorghum vulgare*	2 (1.0%)	-
**Pulses/Seeds**					
	Sarson Beej	Mustard seeds	*Brassica juncea*	59 (30.1%)	94 (62.3%)
	Rehad	Black gram dal	*Vigna mungo* (L.)	49 (25.0%)	56 (37.1%)
	Kulthi	Horsegram	*Macrotyloma uniflorum* (Lam.) Verdc	27 (13.8%)	23 (15.2%)
	Khesari dal	Khesari dal	*Lathyrus sativus* L	9 (4.6%)	-
	Barbatti (bada ghangra)	Cowpea, brown	*Vigna catjang* (L.) Walp	9 (4.6%)	76 (50.3%)
	Chana	Bengal gram, dal	*Cicer arietinum*	2 (1.0%)	1 (0.7%)
	Masoor	Lentil dal	*Lens culinaris*	2 (1.0%)	1 (0.7%)
**Green Leafy vegetable**					
	Sarson patta	Mustard leaves^a^	*Brassica juncea*	68 (34.7%)	101 (66.9%)
	Khesari saag	Khesari leaves^a^	*Lathyrus sativus* L	2 (1.0%)	-
**Other Vegetables**					
	Barbatti (chota ghangra)	Barbatti vegetable	*Vigna catjang* (L.) Walp	29 (14.8%)	55 (36.4%)
	Jhingli	Ridge gourd	*Luffa acutangula* (L.) Roxb	-	53 (35.1%)
	Baingan	Brinjal	*Solanum melongena*	2 (1.0%)	49 (32.5%)
	Tamatar	Tomato	*Solanum lycopersicum*	1 (0.5%)	48 (31.8%)
	Kohda	Pumpkin^a^	*Cucurbita maxima*	-	34 (22.5%)
	Bir Karela	Bitter gourd	*Momordica dioica* Roxb	-	32 (21.2%)
	Kaddu	Bottle gourd	*Lagenaria vulgaris*	1 (0.5%)	29 (19.2%)
	Kokri	Spine gourd	*Momordica dioica* Roxb	-	8 (5.3%)
	Mirch	Chilli	*Capsicum annum*	-	2 (1.3%)
	Phoolgobhi	Cauliflower	*Brassica oleracea* var. botrytis	-	1 (0.7%)
	Matar	Peas	*Pisum sativum*	1 (0.5%)	1 (0.7%)
	Bandhgobhi	Cabbage	*Brassica oleracea* var*. capitata* f*. alba*	-	1 (0.7%)
**Roots and Tubers**					
	Aloo	Potato	*Solanum tuberosum*	52 (26.5%)	86 (57.0%)
	Pyaz	Onion	*Allium cepa*	1 (0.5%)	20 (13.2%)
	Mooli	Radish	*Raphanus sativus*	-	1 (0.7%)
**Fruits**					
	Kela	Banana	*Musa x paradisiaca*	-	1 (0.7%)
	Papita	Papaya^a^	*Carcia papaya*	1 (0.5%)	1 (0.7%)
26	Amda	Mango^a^	*Mangifera indica*	-	1 (0.7%)

^a^Vitamin A rich foods

**Table 4:** List of food items accessed by Santhal households from pond and forests

S. No	Food items	n	%
**Food items accessed from pond (*n* = 118)**			
1	Fish	114	96.6
2	Snail	94	79.7
3	Turtle	33	28.0
4	Crab	7	5.9
**Food items accessed from forests (*n* = 116)**			
1	Wild edible mushrooms	86	74.1
2	Wild edible plants	82	70.7
3	Wild animals	63	54.3
4	Medicinal plants	48	41.4
5	Honey	27	23.3

The main source of drinking water for a majority of households was tube well/hand-pump (63.5%), and a majority of them reported defecating in open field/jungle (91.5%) (Table 2).

### Dietary intake pattern and nutrient intake of Santhal women

Table 5 provides a summary of food group wise intake among Santhal women based on the 2-days 24-h dietary recall. In addition to the cereals, the diet of Santhal women constituted of roots and tubers, other vegetable and pulses. Only a small proportion of women consumed flesh foods (14.2%), fruits (6.2%), and milk and milk products (1.4%).

**Table 5:** Summary of food group intake among Santhal women (*n* = 211)

Food group (grams)	N	%	Mean	SD	Minimum	Maximum
Cereals and millets	211	100.0	256.7	104.7	76	681
Pulses	115	54.5	20.2	19.1	2	89
Green leafy vegetables	88	41.7	54.6	61.6	6	375
Other vegetables	179	84.8	40.1	34.6	1	195
Roots and tubers	197	93.4	67.8	52.9	4	348
Fruits	13	6.2	40.6	34.8	5	128
Milk and milk products	3	1.4	104.4	118.6	20	240
Egg, poultry, meat and fish	30	14.2	31.9	21.7	7	490
Oils and fats	210	99.5	3.73	5.587	0.6	49
Sugar	96	45.5	13.2	14.4	1	73

Based on the 2 day-24 HDR, Table 6 provides a detailed description of mean usual nutrient intakes and the prevalence of nutrient inadequacy. Upon comparing the usual intake of each nutrient with the corresponding EAR, the mean intakes were found to be less than the EAR for all the nutrients.

**Table 6:** Mean of usual nutrient intakes and prevalence of nutrient inadequacy among Santhal women, Jharkhand, India (*n* = 211)

Nutrients	EAR	Mean usual intake	SD	*n* < EAR	%
Energy (kcal/d)	2130	1032	146.3	211	100
Protein (g/d)	36.3	24.7	2.7	211	100
Fat (g/d)	NA	5.2	1.1	NA	NA
Calcium (mg/d)	800.0	60.3	6.2	211	100
Iron (mg/d)	15.0	4.6	0.6	211	100
Zinc (mg/d)	11.0	3.8	0.6	211	100
Vitamin A (µg/d)	390.0	20.3	6.7	211	100
Vitamin C (mg/d)	55.0	23.4	6.1	211	100
Thiamine (mg/d)	1.4	0.3	0.1	211	100
Riboflavin (mg/d)	2.0	0.2	0.03	211	100
Niacin (mg/d)	12.0	5.2	0.6	211	100
Pyridoxine (mg/d)	1.6	0.4	0.1	211	100
Folate (µg/d)	180.0	60.3	11.0	211	100

### Dietary diversity and IF consumption and their contribution to nutrient intake among Santhal women

The mean dietary diversity score obtained was 2.3 ± 0.6 with a median of 2 (range 1, 4) which is lower than the recommended minimum dietary diversity (MDD-W) of more than or equal to five food groups for the WRA group. The mean IF score obtained was 0.9 ± 0.7 with a median of 1.0 (range 0, 3.5).

There was a trend of increasing nutrient intake (all nutrients) with increasing MDD-W score (Table 7). The IF consumption scores were significantly associated with the intake of thiamine and calcium, indicating an increase in intake of these nutrients with a higher intake of indigenous foods.

**Table 7:** Association of IF consumption and MDD-W scores with usual nutrient intakes among Santhal women, Jharkhand, India (*n* = 211)

	**Pearson coefficient 'r'**	
**Nutrients**	**IF consumption scores**	**MDD-W scores**
Energy	0.09	0.25***
Protein	0.09	0.35***
Fat	0.07	0.36***
Thiamine	0.15*	0.27***
Riboflavin	0.05	0.35***
Niacin	0.02	0.28***
Vitamin B6	0.05	0.39***
Folate	0.13	0.49***
Vitamin C	-0.08	0.32***
Vitamin A	-0.12	0.14*
Calcium	0.27***	0.56***
Iron	0.12	0.26***
Zinc	0.08	0.34***

^*^P < 0.05, **P < 0.01, ***P < 0.001

### Micronutrient status and status of inflammatory biomarkers of the Santhal women

Table 8 describes the mean biomarker levels. The mean CRP was found to be 3.1 ± 7.3 mg/L, and the mean AGP was 727.8 ± 259.7 mg/L, with one-fourth of the women reporting elevated levels of both CRP and AGP. While the prevalence of iron deficiency (< 30 ng/ml) based on the unadjusted ferritin values was 65.4% after adjusting the ferritin levels for inflammation using a linear regression model, the prevalence increased to 73%. The prevalence estimates based on the adjusted ferritin and sTfR levels were approximately similar (Table 9). Based on the hemoglobin levels, around 69.6% of the women were iron deficient or anemic, and 5% had severe anemia. Based on RBP levels 28.4% of the women were Vitamin A deficient, and 33.6% of the women had insufficient Vitamin A levels while 37.9% had normal levels.

**Table 8:** Summary of biomarker estimates among Santhal women (*n* = 211)

Biomarker	Mean	SD
CRP (mg/L)	3.1	7.3
AGP-1 (mg/L)	727.8	259.7
Ferritin (ng/ml)	31.3	34.1
Soluble transferrin receptor (µg/ml)	3.3	2.3
Hemoglobin (g/dL) (*n* = 182)	10.6	1.8
Retinol Binding Protein (µmol/L)	1.1	0.8

**Table 9:** Prevalence of micronutrient deficiency and status of inflammation in Santhal women (*n* = 211)

Biomarker	Unadjusted		Adjusted	
	**N**	**%**	**N**	**%**
**Biomarkers of Iron deficiency**				
**Ferritin**				
< 30 ng/ml	138	65.4	154	73
**sTfR**				
≥ 2.07 µg/ml	170	80.6	157	74.4
**Hemoglobin** (g/dL) (*n* = 181)				
Normal (≥ 12)	55	30.4	-	-
Mild anemia (11–11.9)	49	27.0		
Moderate (8–10.9)	68	37.6		
Severe (< 8)	9	5.0		
**Biomarker of Vitamin A deficiency**				
**RBP**			-	-
< 1.05 mmol/L (Vitamin A insufficient)	71	33.6		
< 0.7 mmol/L (Vitamin A deficient)	60	28.4		
**Biomarkers of inflammation**				
CRP/AGP in normal range	80	37.9	-	-
Only CRP elevated	45	21.3		
Only AGP elevated	33	15.6		
Both CRP and AGP elevated	53	25.1		

### Factors associated with the biomarker status of the Santhal women- Determinants of micronutrient status

**Fig. 2:**
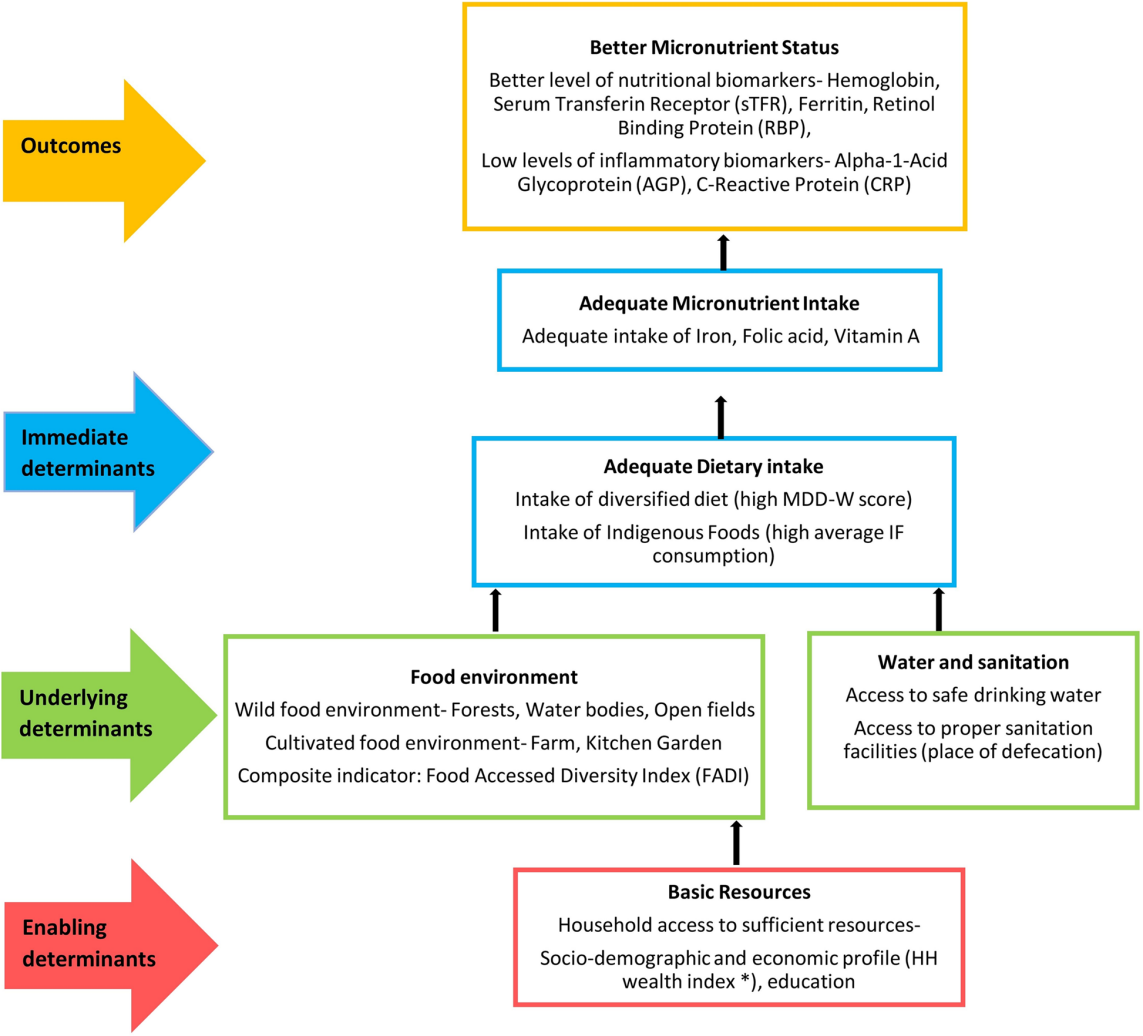
Conceptual framework on enabling, underlying and immediate determinants of micronutrient status of Santhal women, Jharkhand, India adapted from UNICEF conceptual framework on determinants of maternal and child nutrition, 2020. MDD-W, Minimum Dietary Diversity for women. *HH wealth index was generated using household information on type of housing, number of rooms, presence of a separate kitchen, source of electricity and drinking water, ownership of selected assets and monthly expense on food

The following section reports the association between various factors or determinants that contribute to the micronutrient status and the level of biomarkers that reflect the micronutrient status. We analyzed the associations aligned to the conceptual framework (Fig. 2) adapted from the UNICEF conceptual framework on determinants of maternal and child nutrition, 2020 [44]. It explored the association between enabling, underlying and immediate determinants of micronutrient status and the level of nutritional and inflammatory biomarkers as the outcome. The framework facilitated analyzing, organizing, and reporting of the data systematically.

#### A. Enabling determinants of micronutrient status

##### Association between sociodemographic, economic profile and biomarkers

When a one-way ANOVA test or t-test was applied to find the association between sociodemographic variables and the biomarkers, no statistically significant difference was found in group means of the biomarker levels across the wealth quintiles (*p* > 0.05). However, a statistically significant difference between group means of RBP was observed between the two levels of education: primary and below (group 1) and above primary (group 2), wherein the mean RBP was higher in group 1 (mean = 1.19 mmol/L, *p* = 0.008) as compared to group 2 (mean = 0.92 mmol/L, *p* = 0.008). There were no significant differences in mean values of biomarkers among people with different drinking water sources and place of defecation (*p* > 0.05).

#### B. Underlying determinants of Micronutrient status

##### Association between access to different sources of food and the biomarkers

There was no statistically significant correlation between agroforestry diversity index-FADI and the biomarkers as determined by the Karl Pearson correlation coefficient test. Concurrently, when access to different food sources was compared with the biomarkers using t-test, it was found that access to agricultural land was significantly associated with hemoglobin levels (*p* = 0.01), and access to *baari* was significantly associated with the ferritin levels (*p* = 0.02), RBP levels (*p* = 0.02) and level of inflammatory biomarkers, i.e., CRP (*p* = 0.02) and AGP-1a (*p* = 0.01). Access to ponds and forests was significantly associated with the ferritin levels (*p* = 0.004 and *p* = 0.02 respectively for pond and forest).

#### C. Immediate determinants of micronutrient status

##### Association between minimum dietary diversity, indigenous food consumption, specific nutrient intake, and the biomarkers

No statistically significant correlation was found on exploring the correlation between MDD-W scores, indigenous food consumption, and biomarkers (Table 10). However, when the correlation between specific nutrient intake and the biomarkers was explored, it was found that the iron intake positively correlated with the ferritin level (*r* = 0.08, *p* = 0.25) and negatively correlated with the sTfR levels (*r* = -0.07, *p* = 0.32), but the correlation was not significant. The correlation between folate intake and ferritin (*r* = 0.18, *p* = 0.01) and folate intake and sTfR (*r* = -0.14, *p* = 0.03) was statistically significant. Intake of iron, folate, and vitamin C was also positively correlated with the hemoglobin level, with the correlation between folate intake and hemoglobin level being statistically significant (*r* = 0.18, *p* = 0.01) (Table 10). The energy intake was negatively correlated with the level of the inflammatory biomarker CRP (*r* = -0.14, *p* = 0.06), with the correlation being marginally significant.

**Table 10:** Correlation between specific nutrient intake, minimum dietary diversity, indigenous food consumption, and the biomarkers

	**Pearson coefficient 'r'**			
**Variable**	**Ferritin**	**sTfR**	**Hemoglobin**	**RBP**
**MDD-W**	0.05	-0.06	0.08	0.06
**IF consumption**	0.06	-0.1	-0.05	0.06
**Nutrient intake**				
Iron	0.08	-0.07	0.01	-
Folate	0.18**	-0.14*	0.18*	**-**
Vitamin C	0	-0.1	0.07	**-**

^*^P < 0.05, **P < 0.01, ***P < 0.001

#### D. Linear Mixed Effects Regression for assessing factors associated with biomarker status

When the variables that showed significant association in the bivariate analysis (*p* < 0.05) were transferred to the multivariate linear regression model, it was found that women having above primary level of education had 15.3% lower RBP level than women having a level of education of primary or below (*p* < 0.05, 95% CI: -27.82, -1.39). HH access to *baari* was positively associated with the RBP level and negatively associated with inflammatory biomarkers, while access to ponds was positively associated with the ferritin levels. The women who had HH access to *baari* had 18.53% higher level of RBP than those who did not access baari (*p* < 0.05, 95% CI: 2.33, 36.21). Some of the Vitamin A-rich foods accessed from the *baari* included mustard leaves, and pumpkin, accessed by 66.9%, and 22.5% of the HHs respectively as determined by the HH survey (Table 3). The women having HH access to *baari* had 38.12% lower level of CRP (*p* < 0.05, 95% CI: -58.85, -6.01) and 13.06% lower level of AGP-1a than those who did not have access to the same (*p* < 0.05, 95% CI: -22.97, -1.78). Women who had HH access to the pond had 27.12% higher ferritin levels than women who did not have access to the same (*p* =  < 0.05, 95% CI: 1.82,58.57). Some iron-rich food items accessed from ponds included indigenous flesh foods like fish, snail, turtle, etc. (Table 4). Folate intake was positively associated with ferritin and hemoglobin levels, and as the folate intake changed by 1 unit, ferritin level also changed by 1.21% (*p* < 0.01, 95% CI:0.30,2.12) and hemoglobin changed by 0.28% (*p* < 0.05, 95% CI: 0.04,0.52). The folate intake was negatively associated with the sTfR levels and per unit increase in folate intake led to a 0.59% decrease in sTfR levels (*p* < 0.05, 95% CI: -1.19, -0.01).

## Discussion

Our previous work [37, 45] among the indigenous communities of Jharkhand and their food environment suggests that while these communities have access to diverse natural food sources and possess TEK regarding IFs, the utilization of these are sub-optimal. Further, nutritional analysis of specific IFs revealed that these are nutrient-rich with the potential of contributing substantially to the nutrient intakes. Hence, we decided to explore the inter-links between nutritional status and dietary intake, focusing on IF consumption and their relationship with crucial micronutrient biomarkers among the Santhal community in Jharkhand. We also explored the inter-links between the biomarkers and access to different food sources. We specifically chose biomarkers of iron and vitamin A status and of inflammation for our study based on the commonly observed deficiencies in WRA in the region, and guided by the recent recommendations from expert groups [46, 47]. These were analyzed using a multiplex assay to minimize sample requirements and address logistic and technical challenges.

The food environment of the Santhal community consisted of diverse food sources. However, the actual diversity in access and utilization of food from these sources expressed as FADI, an agroforestry diversity index, was low. The dietary intake of the participating women also demonstrated low diversity expressed as MDD-W. Additionally, the mean nutrient intake was less than the EAR for each nutrient, thus reflecting inadequate nutrient intake. The women with higher MDD-W scores had a higher intake of all nutrients, and women -having higher IF scores had a significantly higher intake of thiamine and calcium. The biomarker status of the women revealed that one-fourth of the women had elevated levels of both CRP and AGP. The prevalence of iron deficiency was around 70%. Approximately 33.6% women were Vitamin A insufficient, while 28.4% were deficient. We found that some of the biomarkers were associated with HH access to food sources from the natural environment. The access to *baari* was positively associated with the RBP level and negatively associated with inflammatory biomarkers, while access to ponds was positively associated with the ferritin levels.

A high prevalence of iron deficiency among women in the reproductive age group is a persistent problem in the Indian population (IIPS and ICF 2021). Higher levels of iron deficiency ranging from 36 to 76% [48–51] are also reported among WRA in the various indigenous populations across Indian states, such as Uttarakhand, Kerala, Karnataka, and the north-eastern states. The NFHS-4 data also reports a high level of anemia (75.41%) among indigenous women of the Godda district [52], the region where the present study was conducted. Indigenous populations bear an additional burden of communicable diseases resulting in a high level of infection and inflammation [9]. One of the factors responsible for this could be the lack of sanitary facilities and open defecation, the prevalence of which was high in our study (91.5%). This is a consistent observation across rural India [53]. In this context of low intakes, high nutrient deficiency and increased inflammation, it is essential to note that studies have linked an increase in inflammatory markers with poor iron absorption [54, 55]. So, even if iron supplementation (a significant policy thrust area) and dietary diversification through indigenous foods are promoted in our study population, it is critical to simultaneously address underlying infections and inflammation to improve iron bioavailability and decrease anemia prevalence.

We did not find any significant correlations between the enabling determinants like sociodemographic and economic profiles and micronutrient status. Data from extensive global studies show a variable contribution of sociodemographic factors like education, household assets, healthcare access, sanitation and income quintiles on micronutrient and inflammatory biomarkers [56–58].

Indigenous food sources have demonstrably improved diet quality through dietary diversity [59]. The knowledge and routine consumption of several IFs accessed from the natural food environments is also reported among other indigenous communities worldwide [37, 60, 61]. Many of these foods have substantial quantities of micronutrients like iron, calcium, vitamin A, and zinc [30, 62] which are critical nutrients required for optimum health, especially for vulnerable populations like women and children. Despite the knowledge about diverse food sources, a low diversity in food access and utilization reflected in the low FADI score indicated underutilization of the IFs among Santhals. This has been a consistent observation across our work in the different indigenous communities [37, 61], who demonstrate this paradox of poor and diminishing consumption amidst collective understanding and historical usage of IFs. Other qualitative studies have documented reasons for this underutilization, including impact of climate change on natural food environments, resulting in decreased production and consumption of IFs; easy access to local markets for purchasing foods; and local agricultural organizations' promoting high yield hybrid varieties [61, 63]. However, it was interesting to note that, despite poor access, there is the utilization of some of the natural food sources, reflecting a habitual behavior towards food acquisition and consumption. Many of the food items routinely accessed from *baari, *e.g., mustard leaves and pumpkin, by a substantial number of HHs, have significant amounts of beta-carotene (2619 µg and 1449 µg of beta carotene per 100 g of mustard leaves and pumpkin respectively [64]. The observed correlation of RBP and ferritin levels with access to food from *baari* and ponds justified this premise.

Similarly, food items from ponds such as indigenous flesh foods like fish, snail, turtle, etc. have reasonable amounts of iron (ranging from 1- 2.3 mg/100 g in indigenous fish [45, 61], 2.29 mg/100 g in snail [65] and 311 mg/kg dietary matter in juvenile aquatic turtles [66]. Some studies have reported using biomarkers for testing whether food-related interventions for indigenous communities have had the desired effect [67, 68] concerning expected outcomes. Some have shown that geographical proximity to forest areas is a determinant of biomarker levels such as hemoglobin since location is a surrogate for access to fruits, vegetables, and animal foods from the natural food environment [69, 70]. Other studies have documented a positive impact of IF consumption on biomarker status [71, 72], including reduction of anemia and decrease in inflammation [69]. The latter was also an indirect observation in our study in which women accessing *baari* showed lower levels of anti-inflammatory markers. It is important to note that wild foods, especially green leafy vegetables, fruits, and mushrooms, have anti-inflammatory properties [73–75].

We did not observe any correlation between IF intake and MDD-W with biomarker levels. This finding could be ascribed to the generally low diversity without a significant gradient in intakes among the participants. However, we found some association between nutrient intake and biomarkers e.g., the dietary folate intake correlated negatively with a marker of inefficient erythropoiesis like sTfR. The role of folate as a nutrient necessary for normal hematopoiesis is well established, and since sTfR represents a state of insufficient iron availability, so the inverse correlation between the two is logical from a biological perspective.

Our findings re-affirm the potential of indigenous foods sourced from natural food environments like *baari* and water bodies to contribute to nutritional status. Strategies are required to promote the cultivation of these foods and increase awareness of the nutritional benefits of consuming these foods. The benefits of homestead food production programs coupled with nutrition education have been demonstrated in Bangladesh, Cambodia, Nepal, and the Philippines. They have been reported to increase food diversity and decrease the prevalence of anemia and night-blindness in children and WRA [76]. Projects conducted with William Treaties First Nation in Canada examined prospects of maintaining and using indigenous food systems and promoted traditional food acquisition activities. These included fishing, hunting, cultivating local foods, promoting community knowledge, skill-sharing, and building communal resources [77, 78]. Policy and program strategies incorporating conservation and promotion of indigenous seed varieties may effectively preserve and develop diversity [79]. Many factors affect the feasibility of maintaining/propagating the cultivation of these nutrition-rich foods, including seasonal availability, increasing availability of convenience foods, eroding TEK about their nutritional benefits, and changes in environmental regulations [80, 81]. These should be considered for designing contextual approaches for maximising impact.

### Inference

Our study was conducted within the framework of recommendations from the BOND initiative and similar studies, and estimated appropriately selected nutrient biomarkers concurrently with inflammation markers to explore the impact of indigenous food consumption on biomarker status. Biomarkers were selected to incorporate the impact of multiple variables encompassing geography, access to samples, limited infrastructure, and modified biological responses due to high infection load. The use of a multiplex assay can reduce labor, cost, supplies, and sample volumes. The data also emphasizes the potential of IFs and their utilization in improving micronutrient status since a positive impact was seen on micronutrient biomarkers even with limited utilization. These findings suggest the necessity of including appropriate biomarkers in nutrition and health surveillance programs. Intervention programs need to incorporate strategies to promote routine utilization of diverse foods from the local environment to improve malnutrition in indigenous communities. Strategies such as promoting indigenous plants’ cultivation and rearing of indigenous animals and aquatic species can be incorporated into current agricultural policies. Activities to promote IF production through agricultural extension services can be undertaken. Incorporation of IFs into ongoing food security programs (targeted public distribution system) and food supplementation programs such as supplementary feeding initiatives under ICDS can be done. These activities can be strongly backed by behaviour change communication strategies including nutrition education programs, and activities to promote indigenous food consumption in the habitual diets of Santhal women.

### Study limitations

Due to the cross-sectional nature of the study design, our findings represent only the associations, we cannot draw causal inferences from the same. There is also a possibility of unmeasured confounding factors in this study. Seasonal fluctuations, which are often known to affect nutrient consumption, were not included in the 24- hour DR of the respondent women. There may be some inaccuracy in reported portion size estimates, although standard food recall kits and a portion size estimation flipbook were employed during the survey. The nutrient calculation database used by us, calculates nutrients based on the nutritive value of raw foods using the Indian food composition table. However, it does not consider the nutrient retention factors ascribed to the effect of cooking on the nutrient content. The information on access to different natural food sources was gathered at the HH level, and it may not reflect the actual access/utilization of the women, although we made specific efforts to interview an adult woman during the HH survey. The markers of inflammation chosen by us could be affected by malaria and other infections. While we did ensure that only healthy, afebrile women were chosen as participants; we did not have biomarker-based information on recent malaria infection.

## Supplementary Information

**Additional file 1. **Rawdataset.

## Data Availability

All data generated or analysed during this study are included in this published article.

## Footnotes

1 *TEK refers to the in-depth knowledge base, beliefs and practices possessed by indigenous people about their natural food environment which have been passed down to them through generations by cultural transmission.*

2 *Natural Food environment refers to the subsistence food environment that include wild food (forests, open pastures, aquatic areas *etc*.) and cultivated food environment (fields, closed pastures, gardens from which consumers directly procure food.*
